# The Uterine Appendages—Their Removal by Abdominal Section

**Published:** 1888-01

**Authors:** 


### Editorial.

### *THE UTERINE APPENDAGES: THEIR REMOVAL BY ABDOMINAL SECTION.*

Under various names, operations which in effect produce the menopause artificially, have become of late so common as to rarely claim much attention, unless there are special conditions of interest in reported cases. Nevertheless, the questions involved in this most important surgical procedure, are of sufficient moment, and have such an important bearing upon the longevity of woman withal, as to warrant still frequent discussions in journals as well as societies.

Excision of the ovaries for conditions other than cystic disease is, like its sister, the McDowell operation, essentially an American contribution to the surgical achievements of the nineteenth century; and as ovariotomy has for its most conspicuous exemplar an English surgeon, so has the palm of oöphorectomy passed from American to English hands. It is quite certain that Sir Spencer Wells and Mr. Lawson Tait have made abdominal sections a greater number of times than any other two men in the world. The influence of their work upon surgery in general, as well as upon abdominal surgery in particular, has been of an enduring character.

The history of the operation for the removal of the uterine appendages—its evolution, so to speak—is an interesting one. In 1865, Robert Battey, of Rome, Ga., conceived the idea of extirpating the normally active ovaries, to bring about the menopause in certain cases which had resisted all other means of cure. He did not perform the operation, however, until August 17, 1872. Meanwhile, Hegar at Freiburg, and Lawson Tait at Birmingham, July 27th and August 1st, respectively each, curiously enough practised the operation a few days before Battey, but unbeknown to the latter. Hegar's and Tait's cases were unsuccessful, while Battey's was successful. Both Hegar's and Tait's cases were unpublished—Battey published his in the September following its making, and defended it the next April, before the Georgia Medical Association. Battey contemplated, at this time, only the removal of the ovaries themselves, not particularly for ovarian disease, but to establish the menopause artificially whenever, *for any reason, in any organ,* this should become essential to cure, and only when all other known means had failed. But it was soon discovered that, in some instances, women menstruated even after removal of these organs; so the chief purpose of the operation was thwarted. Thereupon, Tait enlarged the usefulness of the operation by embracing in its scope the Fallopian tubes, believing that they were largely concerned in menstruation; and, further, that they were, even more often than the ovaries, the real seat of the mischief from which relief was sought; so that now the operation is rarely performed, unless the tubes are embraced in the excision. Moreover, the better knowledge of the pathology of tubal disease, and of the ravages produced by it in the surrounding organs and tissues, is leading up to the opinion that, whenever the operation is done at all, it should be done thoroughly, and made to embrace all the annexa, lest any left behind might cause future mischief.

It is curious to note the various changes in name which the operation has undergone. Battey first proposed the designation of normal ovariotomy for his operation. This has received such sweeping condemnation, that it has been dropped altogether. Marion Sims suggested it be called Battey's operation, while Mr. Tait is opposed to naming this, or, for that matter, any other operation, after an individual; and so the names, Battey's operation, Hegar-Tait operation, and Tait's operation, have been gradually disappearing from the literature of the subject. Oöphorectomy expresses in a word, with a tolerable correctness in derivation and meaning, the original operation of Battey; and when the tubes are also to be excised, salpingo-oöphorectomy is adopted by some operators to express the surgical act. But, if hyphenized proper names are becoming popular in the super-refined realm of the fashionable world, not so with medical or surgical phrases; here the trend is towards simplicity of thought and expression. Spaying and castration are both atrociously shocking to the ears of the delicate patients, who often seek restoration to health through the justifiable mutilation of their sexual organs; hence, these terms can never be made to fit kindly to the environments which naturally belong to the operation.

Taken all in all, therefore, the expression, "The removal of the uterine appendages by abdominal section", even though it be open to the objection of lengthiness, would seem to best convey the real idea of the operation as at present practiced, and is coming into quite general use in its literature.

The operation has now passed through the period of trial and criticism, and the verdict of the profession is that it has a field, and that its judicial employment in appropriate cases is to be approved on grounds of humanity and science, as affording the safest, surest, and speediest relief in many diseases of the uterine appendages, otherwise incurable.

